# Transcriptional Regulation of NK Cell Development by mTOR Complexes

**DOI:** 10.3389/fcell.2020.566090

**Published:** 2020-11-10

**Authors:** Chao Yang, Subramaniam Malarkannan

**Affiliations:** ^1^Laboratory of Molecular Immunology and Immunotherapy, Versiti Blood Research Institute, Milwaukee, WI, United States; ^2^Department of Microbiology and Immunology, Medical College of Wisconsin, Milwaukee, WI, United States; ^3^Department of Medicine, Medical College of Wisconsin, Milwaukee, WI, United States; ^4^Department of Pediatrics, Medical College of Wisconsin, Milwaukee, WI, United States

**Keywords:** NK cell development, mTORC1, mTORC2, raptor, rictor

## Abstract

The mechanistic target of Rapamycin (mTOR) is essential for multiple cellular processes. The unique roles of mTOR complex 1 (mTORC1) or mTOR2 in regulating immune functions are emerging. NK cells are the major lymphocyte subset of innate immunity, and their development and effector functions require metabolic reprogramming. Recent studies demonstrate that in NK cells, conditionally disrupting the formation of mTORC1 or mTOR complex 2 (mTORC2) alters their development significantly. Transcriptomic profiling of NK cells at the single-cell level demonstrates that mTORC1 was critical for the early developmental progression, while mTORC2 regulated the terminal maturation. In this review, we summarize the essential roles of mTOR complexes in NK development and functions.

## Introduction

The identification and characterization of the mechanistic target of Rapamycin (mTOR) are closely associated with the discovery of Rapamycin (Sirolimus). In 1964, a Canadian expedition discovered Rapamycin from the soil samples from the South Pacific island of Rapa Nui (Easter Island), which possessed high anti-fungi, anti-tumor, and immunosuppressive effects (Vezina et al., 1975; Martel et al., 1977; Eng et al., 1984). Georges Nogrady, a microbiologist, collected the soil samples from different parts of the Easter Island to search for why the barefoot islanders did not get tetanus. In 1975, it was found that the *Streptomyces hygroscopicus* produced an anti-fungal compound that was able to inhibit the growth of *Candida albicans*, *Microsporum gypseum*, and *Trichophyton granulosum* (Sehgal et al., 1975; Vezina et al., 1975). In 1982, the immunosuppressive and anti-tumor functions of Rapamycin were discovered (Eng et al., 1984). Chung et al. (1992) found that Rapamycin forms complexes with peptidyl-prolyl isomerase FKBP1A (also known as FKBP12) to mediate its anti-proliferative functions (Kuo et al., 1992). The genetic screening of Rapamycin-resistance led to the identification of the TOR/DRR gene. In 1994, the mTOR-FKBP12 complex in mammalian cells was identified (Brown et al., 1994; Sabatini et al., 1994; Sabers et al., 1995). For the past 25 years, numerous researchers have worked on mTOR protein and defined its essential role in cell growth and functions (Sabatini, 2017).

**FIGURE 1:**
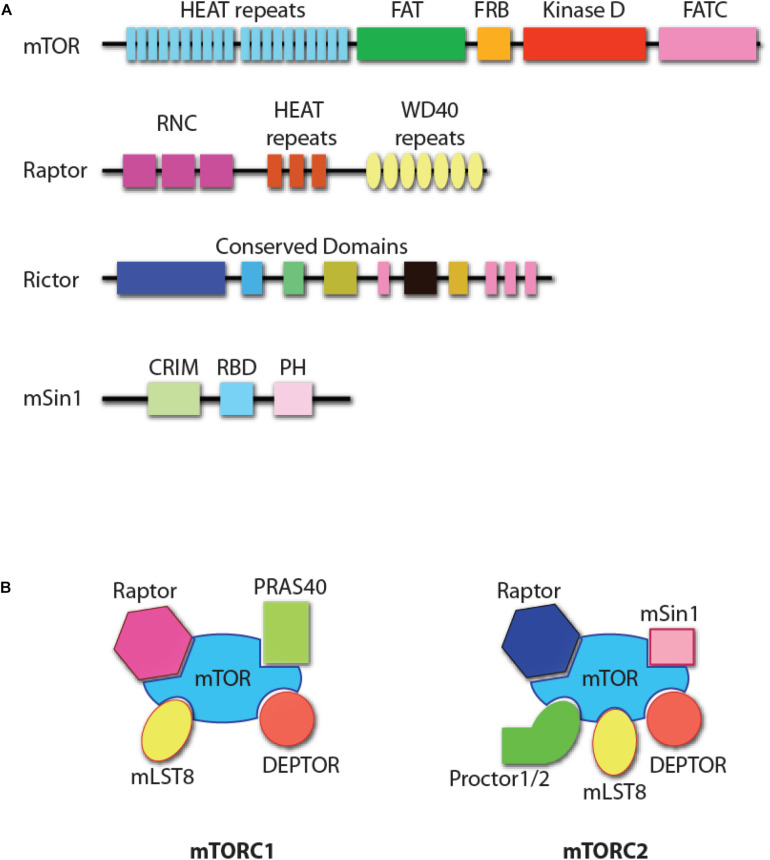
mTOR complexes. **(A)** Protein domain structure of mTOR, Raptor, Rictor, and mSin1. HEAT repeats, tandem repeats of the anti-parallel α-helices important for protein–protein interaction; FAT, a domain found common in PIK-related kinases subfamilies FRAP, ATM, and TRRAP subfamilies; FRB, FKBP12-rapamycin-binding (FRB) domain; FATC, FAT C-terminus; RNC, Raptor N-terminal conserved domain; WD40 repeats, tandem repeats of a structural domain composed about 40 amino acids terminating with tryptophan and aspartic acid (WD); CRIM, conserved region in the middle; RBD, Ras-binding domain; PH, pleckstrin homology domain. The functional domains of Rictor are unknown, with some structure domains that are conserved among species. **(B)** The composition of mTOR complex 1 (mTORC1) and mTOR complex 2 (mTORC2). DEPTOR and mLST8 are the shared components of the two complexes. Raptor and PRAS40 are unique to mTORC1, while Rictor, mSin1, and Protor1/2 are unique to mTORC2.

Mechanistic target of Rapamycin is an evolutionarily conserved 289 kDa serine/threonine kinase of phosphoinositide 3-kinase-related protein kinases (PIKK, Figure 1A) (Saxton and Sabatini, 2017). mTOR forms two structurally distinct complexes, mTOR complex 1 (mTORC1) and mTOR complex 2 (mTORC2) with unique substrate specificities and functions (Saxton and Sabatini, 2017). mTORC1 consists of mTOR, Raptor (regulatory protein associated with mTOR), mLST8 (mammalian lethal with Sec13 protein 8), PRAS40 (proline-rich Akt substrate of 40 kDa), and DEPTOR (DEP domain-containing mTOR interacting protein, Figure 1B) (Saxton and Sabatini, 2017). Genetic studies have demonstrated that Raptor is the essential component in the formation of mTORC1 (Hara et al., 2002; Kim et al., 2002). mTORC2 comprises mTOR, Rictor (rapamycin-insensitive companion of mTOR), mSin1 (mammalian stress-activated protein kinase interacting protein 1), Protor1/2 (protein observed with Rictor-1/2), mLST8, and DEPTOR (Figure 1B) (Saxton and Sabatini, 2017). Both Rictor and mSin1 are essential for the formation of mTORC2 (Jacinto et al., 2004, 2006; Sarbassov et al., 2004; Frias et al., 2006; Yang et al., 2006).

There are five major structural domains of mTOR. This includes the tandem HEAT domain, the FAT (FRAP, ATM, and TRRAP, all PIKK family members) domain, the FRB (FKBP12/rapamycin binding) domain, and the FATC (FAT C-terminus) domain (from N-terminus to C-terminus, Figure 1A) (Yang and Guan, 2007). The tandem HEAT domain mediates the protein–protein interaction between mTOR and Raptor, and the homodimerization of mTORC1 (Yip et al., 2010; Aylett et al., 2016; Baretic et al., 2016). Raptor contains a conserved domain in the N-terminus and seven WD40 repeats, which may facilitate the interactions with mTOR or mTORC1-associated proteins. Rictor is also predicted to contain HEAT repeats and WD40 domains (Zhou et al., 2015). Pleckstrin homology (PH) domains present in Rictor help mediate signal transduction and subcellular localization (Zhou et al., 2015). Another mTORC2 component, mSin1, has a central conserved domain, a Ras-binding domain, and a C-terminal PH domain (Schroder et al., 2004, 2007). The PH domain of mSin1 interacts with the kinase domain of mTOR (Liu et al., 2015). The different composition of the accessory proteins determines that only the FRB domain in mTORC1, but not mTORC2, is accessible to the FKBP12/Rapamycin complex. This results in the inhibition of mTORC1, but not mTORC2. However, prolonged incubation of cells with Rapamycin does inhibit mTORC2 function primarily due to compromised formation of mTORC2, as rapamycin-bound mTOR protein cannot be incorporated into mTORC2 (Sarbassov et al., 2006). Significant progress has been made in defining the essential roles played by mTOR complexes in NK cells (Donnelly et al., 2014; Marcais and Walzer, 2014; Marcais et al., 2014, 2017; Nandagopal et al., 2014; Yang et al., 2016, 2018). In this review, we summarize the relevance of these findings in the context of NK cell development and functions.

## γ_C_-Utilizing Cytokine Receptors Link mTORC1 to NK Cell Development

**FIGURE 2:**
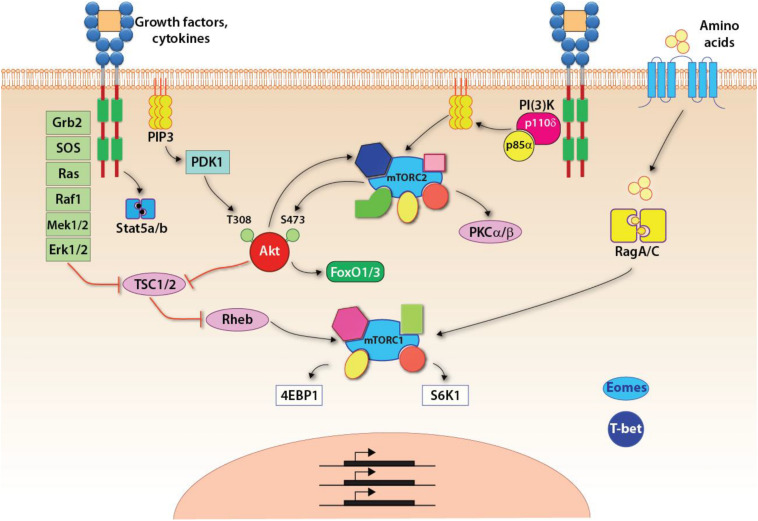
Signaling pathways up and downstream of mTOR complexes. Upon growth factors- or cytokine-mediated activation, three major pathways are initiated: the Jak-Stat5 pathway, the PI(3)K-Akt-mTOR pathway, and the Ras-Raf-Mek-Erk1/2 MAPK pathway. Specific to the mTORC1 pathway, the tuberous sclerosis complex (TSC) functions as a GTPase-activating protein (GAP), which inhibits the activity of Rheb, a small GTPase absolutely required for the activation of mTORC1. Thus, TSC is a central negative regulator of mTORC1 signaling. The activated Akt or Erk, downstream of PI(3)K-Akt or MAPK pathway, respectively, phosphorylates TSC and inhibits its GAP activity resulting in the activation of mTORC1. In addition, amino acids are required for anchoring mTORC1 on the lysosomal membrane where Rheb locates. This is achieved through the RagA/C.

NK cells develop in the BM (Kondo et al., 1997). Common lymphoid progenitors (CLPs) give rise to the early innate lymphoid progenitors (EILPs) that differentiate into all three ILC lineages and conventional NK cells (Yang Q. et al., 2015). Development of NK cell is regulated by multiple common-gamma chain-containing cytokine (γc, CD132) receptors that utilize PI(3)K, as a major signaling link to mTOR complexes (Figure 2). There are five members in the γc family (IL-2, IL-4, IL-7, IL-15, and IL-21), all transduce their signaling via PI(3)K and thereby mTOR complexes (Boulanger and Garcia, 2004). The distinctions among the γc chain receptor family come from the unique α-chain utilization and the differential activation of unique STATs. The earliest indication of the commitment to the NK lineage is defined by the expression of the IL-15/IL-2 receptor β chain (CD122) (Rosmaraki et al., 2001). Thus, the initial commitment of NK cells is tightly associated with the optimal functions of mTOR complexes.

Both IL-15 and IL-2 can bind to the receptor complex formed by IL-2/15R and γc chain and transduce the signals primarily via PI(3)K (Bamford et al., 1994; Giri et al., 1994; Grabstein et al., 1994). The essential role of γc revealed by the fact that individuals with common gamma chain mutations develop severe immunodeficiency with nearly complete loss of NK cells (Noguchi et al., 1993). IL-2 binds to the heterotrimeric receptors composed of CD122, CD132, and IL-2Rα (CD25) with high affinity (Rickert et al., 2005; Stauber et al., 2006). In contrast, IL-15 has a similar high-affinity binding with IL-15Rα (CD215) alone (Giri et al., 1995). Among these two cytokines, IL-15 has been shown to be critical for NK cell development and effector functions (Becknell and Caligiuri, 2005; Marcais et al., 2013). A low dose of IL-15 is sufficient to sustain survival signaling in NK cells, while a high dose of IL-15 promotes NK cell proliferation and effector molecules expression (Marcais et al., 2013, 2014). Through the genetic ablation of individual cytokine members or receptors, the importance of IL-15 in the development of NK cells is now well established. CD132 deficiency in mice also eradicates the NK compartment (DiSanto et al., 1995). Genetic deletion of *Il15* or *Il15ra* but not *Il2* results in a similar loss of mature NK cells seen in the γc chain-deficient mice, firmly establishing the central role of IL-15 in the development of NK cells (Lodolce et al., 1998; Kennedy et al., 2000; Vosshenrich et al., 2005). As the expression of IL-15 and IL-15Rα occurs in the same cells, this high-affinity interaction results in membrane-bound IL-15/IL-15Rα complex (Giri et al., 1995). Thus, the IL-15 signaling initiates through the trans-presentation of IL-15 anchored by IL-15Rα in the neighboring cells to the IL-2/15Rβ/γc complex-bearing cells (Dubois et al., 2002; Mortier et al., 2008). The role of mTOR in the IL-15-dependent developmental progression and priming of NK cells are well established (Marcais et al., 2014; Nandagopal et al., 2014; Mah et al., 2017), which we discuss in the following section.

## IL-15R Utilizes PI(3)K to Activate mTORC1 in NK Cells

Three major pathways, including the Jak1/3-Stat5a/b, the PI(3)K-mTOR, and the MAPK (Boyman and Sprent, 2012), exist downstream of IL-15R. The first pathway involves Jak-mediated activation of Stats. Upon IL-15 binding, IL-2/15Rβ/γc complex recruits Jak1 and Jak3 (Boussiotis et al., 1994; Miyazaki et al., 1994; Russell et al., 1994; Zhu et al., 1998). Jak1 and Jak3 phosphorylate Tyr392 and Tyr510 at the H-region, which serves as critical docking sites for downstream functional proteins, including transcription factors Stat5a and Stat5b (Truitt et al., 1994; Fujii et al., 1998). Serving as the proximal signaling module, it is not surprising that the loss of Jak3 results in the absence of NK cells in mice (Park et al., 1995). Downstream of Jak1/3, both Stat5a and Stat5b are essential to maintain the homeostasis of NK cell pool in mice. Thus, Stat5b deficiency results in a severe loss of NK cells (Imada et al., 1998; Eckelhart et al., 2011). Recent work has demonstrated a correlation between the absolute number of splenic NK cells and the copy number of Stat5a/b. The second pathway initiated downstream of the IL-15 receptor involves the PI(3)K-mTOR axis. Class IA PI(3)Ks include p100α, p100β, and p100δ, that are recruited to the membrane via the regulatory subunit PI(3)K-p85α (Okkenhaug, 2013). Both p100β and p100δ are essential for the development of NK cells, although the detailed mechanisms require further investigations (Kim et al., 2007; Tassi et al., 2007; Guo et al., 2008). Downstream of PI(3)K, mTOR initiates the functions of distinct transcription factors that govern the development and functions of NK cells (Marcais et al., 2014).

The activation of mTORC1 is tightly controlled by the availability of nutrients as mTORC1-mediated anabolism requires sufficient energy and metabolites for the synthesis of macromolecules. Growth factor and mitogen-defendant pathways are potent stimuli for the activation of mTORC1 (Figure 2). A central regulatory mechanism that governs the activation of mTORC1 through pro-growth signaling is the heterotrimeric tuberous sclerosis complex (TSC) comprising TSC1, TSC2, and TBC1D7 (Dibble et al., 2012). TSC functions as a GTPase activating protein (GAP) that inhibits the activity of small GTPase Rheb, which binds and activates mTORC1 (Inoki et al., 2003; Tee et al., 2003; Long et al., 2005). Both PI(3)K-PDK1-Akt and the third pathway downstream of IL-15R involving MAPK promote the phosphorylation of TSC2 and inhibit the function of TSC (Inoki et al., 2002; Manning et al., 2002; Roux et al., 2004; Ma et al., 2005). The inhibition of TSC allows the GTP-bound Rheb to activate mTORC1. Recent studies have shown that TSC1, a negative regulator of mTORC1 and mTORC2, was not required for the terminal maturation and survival of NK cells (Yang et al., 2016). Also, the cytotoxic potentials and the ability to generate inflammatory cytokines were intact in the absence of TSC1. However, TSC1 was needed to limit the exhaustive proliferation of developing immature NK (iNK) cells downstream of IL-15R. Exposure of iNK cells to IL-15 significantly upregulated the expression of TSC1. These findings validate the essential role played by TSC1 in regulating the functions of mTORC1 in iNK cells.

Besides the cytokine-mediated stimuli, sensing the levels of amino acids in the cytoplasm also activates mTORC1. The presence of amino acids in the cytosol anchors mTORC1 to the lysosomal membrane through the heterodimeric Rag GTPase (Kim et al., 2008; Sancak et al., 2008). This enables activation of mTORC1 by Rheb, which is also present in the lysosomal membrane (Menon et al., 2014). Under cellular stress, the activation of mTORC1 is suppressed mainly through AMPK-mediated phosphorylation and activation of TSC2 or direct phosphorylation of Raptor (Shaw et al., 2004; Gwinn et al., 2008). Indeed, a higher level of KLRG1 receptor expression induced the activation of AMPK that negatively regulated NK cell effector functions (Muller-Durovic et al., 2016). Inhibition of Rag GTPases blocks mTORC1-mediated functions (Kalender et al., 2010). As activation of mTORC1 promotes NK cell growth and proliferation, the downstream targets of mTORC1 are often involved in the syntheses of proteins, lipids, and nuclear acids. The best-characterized downstream targets of mTORC1 are 4EBPs and S6K1, both of which are highly involved in the protein synthesis. The 5′cap-dependent mRNA translation requires the formation of the eIF4F complex (Merrick, 2004). One vital component in the eIF4F complex is eIF4E, which recognizes the 5′-cap of mRNA (Merrick, 2004). 4EBPs bind eIF4E and inhibit the assembly of the eIF4F complex, which in turn inhibits the 5′cap-dependent mRNA translation (Richter and Sonenberg, 2005). mTORC1 sequentially phosphorylates multi-sites on 4EBPs and dissociate 4EBPs from eIF4E to promote 5′cap-dependent mRNA translation (Brunn et al., 1997; Gingras et al., 1999). Studies have also demonstrated that the mTORC1-4EBPs axis mostly affects a group of mRNAs named 5′-TOP mRNA, which contains the 5′-terminal oligopyrimidine motif (Meyuhas, 2000; Thoreen et al., 2012). The majority of protein products translated from the 5′-TOP mRNA are involved in protein synthesis (Meyuhas, 2000).

Another well-established mTORC1 target, S6K1, also regulates protein translation. mTORC1 phosphorylates the hydrophobic motif of S6K1 at Thr389 that results in conformational changes leading to the phosphorylation by PDK1 (Pearson et al., 1995; Alessi et al., 1998; Pullen et al., 1998). The phosphorylated and activated S6K1 promotes 5′cap-dependent mRNA translation by phosphorylating eIF4B, a critical component of eIF4F complex (Holz et al., 2005). S6K1 can also phosphorylate and promote the degradation of PDCD4, a negative regulator of mRNA translation (Dorrello et al., 2006). Besides promoting protein synthesis, S6K1 also phosphorylates and promotes degradation of PDCD4, a negative regulator of mRNA translation (Dorrello et al., 2006). Besides promoting protein synthesis, S6K1 also phosphorylates and activates sterol regulatory element-binding protein 1 and 2 (SREBP and SREBP2), which promotes *de novo* lipid synthesis that is critical for cell growth and proliferation (Duvel et al., 2010). In addition to S6K1, mTORC1 has also been shown to promote the SREBP pathway through the regulation of lipin 1 (Peterson et al., 2011). In addition to lipid metabolism, recent studies have established the mTORC1-S6K1 axis in regulating the *de novo* purine and pyrimidine synthesis (Ben-Sahra et al., 2013, 2016; Robitaille et al., 2013). How mTORC1 regulates to achieve optimal NK cell development needs to be explored in the future.

## IL-2R and IL-15R Initiate mTORC2 Activation in NK Cells

The precise molecular mechanism by which mTORC2 regulates NK cell development and functions and the interplay between mTORC1 and mTORC2 in NK cells are under active investigations. Recent studies have established the requirement of mTORC2 function in NK cell development (Marcais and Walzer, 2014; Marcais et al., 2014, 2017; Yang et al., 2018, 2020). Under homeostatic conditions, both mTORC1 and mTORC2 are activated at relatively higher levels in iNK cells compared to mature NK cells (Marcais et al., 2017). Unlike the specific inhibition of mTORC1 by Rapamycin, currently, there is no mTORC2-specific inhibitor, which hinders the study of mTORC2. The inhibition of mTORC2 activity by wortmannin, a specific PI(3)K inhibitor, has led to the speculation that mTORC2 is downstream of the PI(3)K pathway (Sarbassov et al., 2005), which implies its activation downstream of γc-chain cytokine receptors such as IL-15R (Figure 2). Liu et al. (2015) have found that the PH domain of mSin1 interacts with the kinase domain of mTOR and inhibits the kinase activity of mTORC2. The PtdIns(3,4,5)P_3_ generated by PI(3)K interacts with the PH domain and releases the inhibition of the kinase domain of mTOR. Besides this allosteric activation of mTORC2, Akt, downstream of PI(3)K, phosphorylates mSin1 at Thr86 site and promotes the activation of mTORC2 (Yang G. et al., 2015). Whether this phosphorylation also relieves the inhibition mediated by the PH domain remains unknown. Also, Zinzalla et al. (2011) have reported that PI(3)K signaling promotes the association of mTORC2 with the ribosomes, and this spatial regulation also induces the activation of mTORC2, although the mechanism is unknown.

The well-characterized downstream target of mTORC2 is Akt. The phosphorylation of Akt at the Ser473 site is exclusively mediated by mTORC2 and therefore is the standard measurement of mTORC2 activity (Frias et al., 2006; Guertin et al., 2006). Although Akt is upstream of mTORC1, phosphorylation of Ser473 on Akt does not seem to affect the activation of mTORC1 (Guertin et al., 2006). Mechanistically, phosphorylation of Thr308 mediated by PDK1, instead of Ser473, is critical for the kinase activity of Akt (Alessi et al., 1997; Hill et al., 2001), while the Ser473 phosphorylation seems to dictate the substrate specificity of Akt (Jacinto et al., 2006). Akt-mediated phosphorylation of FoxO1/FoxO3a requires mTORC2, and this axis is vital in regulating apoptosis and proliferation (Guertin et al., 2006; Jacinto et al., 2006). mTORC2 also phosphorylates PKCα that regulates cytoskeletal remodeling (Jacinto et al., 2004; Sarbassov et al., 2004). Subsequently, more members of the PKC family were found to be the targets of mTORC2 and involved in the regulation of cytoskeleton (Gan et al., 2012; Li and Gao, 2014). This reveals why perturbing the mTORC2 pathway has a significant negative impact on the cytotoxic potentials of NK cells that depends on cytoskeletal remodeling and vesicular trafficking.

The knowledge related to the interplay between mTORC1 and mTORC2 in NK cells is emerging. Interestingly, mTORC1 can indirectly influence the activation of mTORC2 through a negative feedback loop through S6K1-Grb2 or S6K1-IRS1 axis to inhibit insulin-mediated PI(3)K activation (Harrington et al., 2004; Shah et al., 2004; Hsu et al., 2011; Yu et al., 2011). Other studies have shown that mTORC1 positively regulates mTORC2 function by sustaining IL-15R-mediated signaling. In contrast, mTORC2 represses mTORC1-mediated effector functions of NK cells by repressing STAT5-mediated SLC7A5 expression (Wang et al., 2018). Further studies are warranted to determine both the independent and interdependent functions of mTORC1 and mTORC2 in NK cells.

## mtor-Dependent Metabolic Reprogramming in NK Cells

Mechanistic target of Rapamycin complex 1-mediated glycolysis has been related to the function of NK cells (Donnelly et al., 2014; Mah et al., 2017). Marcais et al. (2014) demonstrated the critical role of mTOR in the proliferation and granzyme B expression-mediated by IL-15 during viral infection using an NK cell-specific *Mtor* knockout mouse. In addition, the development of NK cells is significantly impaired in these mice (Marcais et al., 2014). Most of the functional defects have been attributed to mTORC1 due to comparable impairments induced by rapamycin (Donnelly et al., 2014; Nandagopal et al., 2014). TGF-β suppresses the effector functions of NK cells through inhibition of mTORC1 (Viel et al., 2016). Earlier studies have shown that IL-2-mediated stimulation of NK cells in the presence of TGF-β significantly reduced levels of oxidative phosphorylation, maximal respiration, and glycolytic capacity; but, not glycolysis (Viel et al., 2016). Presence of TGF-β also reduced the expression of CD69, CD71, IFN-γ, and granzyme B. Treatment of these cells with TGFβR1 inhibitor reversed these effects except granzyme B. Lack of the TGFβR2 in NK cells reduced the levels of granzyme B in NK cells, validating the link between TGF-β and mTORC1. It is predicted that the effect of TGF-β is mediated by a non-Smad pathway that involves PI(3)K/Akt signaling. Besides cytokines-mediated signaling, mTOR is also activated downstream of NK cell activating receptors, and its activity is associated with the responsiveness of the cells (Marcais et al., 2017).

The link between mTORs and mitochondrial functions is emerging (Cong et al., 2018; Summer et al., 2019). mTOR-mediated metabolic reprogramming is linked to mitochondrial and cell respiration (Zheng et al., 2019). The presence of NK cells within the tumor microenvironment has been well-established (Habif et al., 2019). However, the mechanistic basis for their inaction against the tumor cells is yet to be understood. Recent studies have shown that a hypoxic condition established by the tumor initiates and sustains the activation of mTOR-GTPase dynamin-related protein-1 (mTOR-Drp1) (Zheng et al., 2019). Phosphorylation is an essential event for the mitochondrial pro-fission function of Drp1. This study revealed that a hypoxic environment leads to sustained mTOR activation via AKT-TSC1/2 signaling in human NK cells. This hyperactivation of mTORC1 augmented the phosphorylation and activation of mTOR-Drp1, leading to mitochondrial fragmentation and failure in their anti-tumor effector functions. The sustained activation of Drp1 was mediated by an mTORC1/4EBP1-dependent translation and expression of mitochondrial fission process-1 (MTFP1) protein. MTFP1 mediates the phosphorylation of Drp1 potentially via a retrograde signaling pathway and the respective kinases. Recruitment of Drp1 to the mitochondria leads to its fragmentation and prevents their branching and hyperfusion (Morita et al., 2017). Thus, persistent activation of mTORC1 due to hypoxia can lead to a failure of NK cells. While these findings identify novel therapeutic targets, additional studies can help to define the mechanisms of NK cell impairments under non-hypoxic conditions.

## Transcriptional Regulation of NK Cell Development by mTORC1 and mTORC2

**FIGURE 3:**
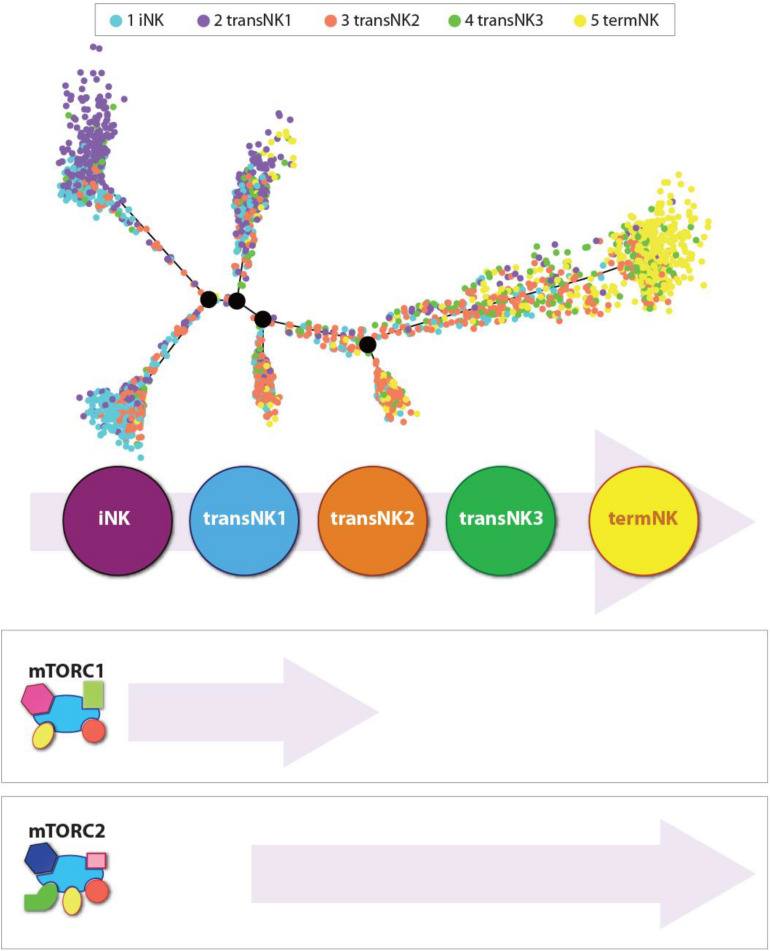
Independent roles of mTORC1 and mTORC2 during the development of NK cells. Distribution of all five NK clusters along the pseudotime trajectory from the bone marrow of wild-type C57BL/6 mice. The relative maturity of the developmental trajectory is displayed across pseudotime. Distribution of each NK clusters along the pseudotime trajectory. Data presented are adapted from Yang et al., 2020.

The evolving transcriptome of developing mouse and human NK cells has recently deciphered (Crinier et al., 2018; Yang et al., 2018, 2019, 2020). These studies provide the opportunity to identify the transcriptional activation or repression during NK cell ontology. Generation of *Ncr1*^*iCre*^-based conditional knockout mice for Raptor (*Rptor*; *Rptor^*fl/fl*^Ncr1^*Cre/WT*^*) and Rictor (*Rictor*; *Rictor^*fl/fl*^Ncr1^*Cre/WT*^*)-encoding genes provided unique opportunities to perform focused analyses of NK cells (Figure 3). These murine models, combined with single-cell RNA sequencing (scRNA-seq) technology, have allowed investigators to define the unique role of mTOR complexes in the transcriptional regulation of NK cell development and functions (Yang et al., 2018). Single-cell RNA-sequencing technology has provided us an unprecedented insight into the transcriptomic profiles of NK cell heterogeneity and development (Crinier et al., 2018; Yang et al., 2019; Dogra et al., 2020). Using this approach, the transcriptional regulations mediated by mTORC1 and mTORC2 were recently determined (Yang et al., 2020).

In the bone marrow of the WT mice, single-cell transcriptome analyses reveal five distinct NK developmental subsets. They are an iNK cluster, three transitional NK (transNK1, transNK2, and transNK3) clusters, and a terminally mature NK (termNK) cluster. The iNK cluster represented the most immature stage with high expression of *Cd27* and low expression of *Itgam* (CD11b) and Ly49s (*Klra1/3/4/7/8/9*), and high expression of *Ltb*, *Thy1*, *Cd3d/g*, and *Cd7*. In contrast, the termNK cells were defined by the low expression of *Cd27* and high expression of *Itgam Gzma*, *Gzmb*, and *Klrg1*. The transNK1 cluster possessed a high expression of genes encoding proteins involved in ribosomal biogenesis, including ribonucleoproteins (*Nop10*, *Nhp2*, *Gar1*, *Npm1*, *Npm3*), RNA modification enzymes (*Mettl1*, *Ddx21*, *Fbl*), and GTPase related to nucleocytoplasmic transport (*Ran*, *Ranbp1*). Notably, some of the major metabolic pathways that are regulated by mTORC1, including glycolysis, oxidative phosphorylation, and fatty acid metabolism, were upregulated in the transNK1 cluster. This confirmed a direct role of mTORC1 in the early developmental stages of NK cells.

Lack of Raptor (mTORC1) resulted in significant NK cell developmental defects as the early stages (Yang et al., 2018). Conditional ablation of mTORC1 by the deletion of Raptor resulted in the blockade of NK cell development at the CD27 single-positive stage in the BM of *Rptor^*fl/fl*^Ncr1^*Cre/WT*^* mice. mTORC1 is required for the expression of Eomesodermin (Eomes) and the transition from CD27 SP to DP NK stage, while mTORC2 is required for the terminal CD11b SP NK cell maturation through the mTORC2-Akt^*S*473^-FoxO1 axis (Yang et al., 2018). Eomes and T-bet belong to the T-box family of TFs, which control multiple aspects of NK cell development and maturation (Townsend et al., 2004; Intlekofer et al., 2005; Gordon et al., 2012). Both contain highly conserved DNA-binding domains, indicating they bind to the same transcription factor-binding motifs. However, the interacting partners of Eomes and T-bet vary (Naiche et al., 2005; Zhang et al., 2018). IL-15 receptor, which activates the mTORC1 complex, plays an essential role in the early commitment and development of NK cells by promoting the transcription of E4BP4 (Kamizono et al., 2009). In turn, E4BP4 induces the expression of Id2 and Eomes, two essential transcription factors for the early NK cell development (Yokota et al., 1999; Boos et al., 2007; Gordon et al., 2012; Male et al., 2014; Delconte et al., 2016). The single-cell transcriptomic profiles of NK cells from *Rptor* cKO mouse reveal that the iNK cell stage (CD27 single positive) failed to progress into the termNK cells (CD11b single positive).

Reduced NK cell number in the periphery, reduced steady-state proliferation, and impaired migration *in vitro* demonstrate that the disruption of homeostatic NK cellularity is disrupted in *Rptor* cKO mice. Moreover, loss of mTORC1 significantly impaired NK cell maturation, as demonstrated by the accumulation of CD27 SP population and reduced DP and CD11b SP populations. This defect may directly contribute to the accumulation of NK cells in the BM, as they gradually obtain migratory capacity following CD11b expression (Mayol et al., 2011). Despite these findings, *Ncr1*^*Cre*^-mediated deletion of *Pdpk1* or *Tsc1* did not show any defects in NK cell development (Yang M. et al., 2015; Yang et al., 2016). This demonstrates that following Ncr1 expression, mTORC1 is activated through an alternative mechanism instead of the canonical PI(3)K-PDK1-Akt-TSC1/2-mTORC1 pathway. mTORC1 regulates protein translation through various mechanisms. One of which directly affects the translation of proteins comprising the translational machinery such as eIFs, and ribosomal proteins (Meyuhas, 2000; Thoreen et al., 2012).

**FIGURE 4:**
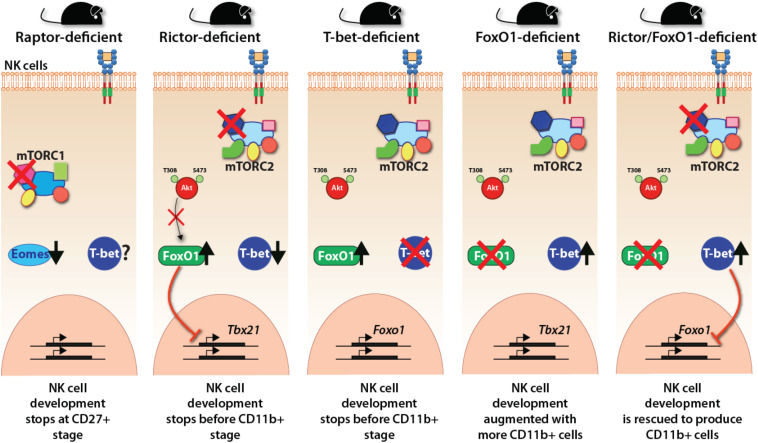
Transcriptional regulations downstream of mTOR complexes. The essential regulation of crucial transcription factors downstream of mTORC1 and mTORC2 are shown employing the observations made with distinct knockout mice. mTORC1 regulates the expression of Eomes and thereby the early developmental stages of NK cells. mTORC2 regulates the reciprocal repressive activity between FoxO1 and T-bet. FoxO1 represses the expression of T-bet, thereby maintaining the immature gene signature. T-bet suppresses the expression of FoxO1 to make the NK cells progress to the terminally mature NK cell stage.

In contrast, mTORC1, lack of mTORC2 in *Rictor^*fl/fl*^ Ncr1^*Cre/WT*^* mouse resulted in a blockade that did not allow CD27/CD11b double-positive NK cells to progress into terminally mature CD11b single-positive stage. scRNA-seq data demonstrate that in the absence of Rictor, NK cells possessed a high expression of upregulated genes of the iNK cluster, confirming this blockade (Figure 4). Importantly, lack of functional mTORC2 resulted in the significant upregulation of Forkhead transcription factors of the O class-1 (FoxO1). This family contains a winged-helix DNA-binding domain and the forkhead domain (Obsil and Obsilova, 2008). Earlier studies have shown that among the four members of this family (FoxO1, 3, 4, and 6), FoxO1 is highly expressed in NKPs and iNKs compare to mNKs. FoxO3 is expressed at all stages of NK cell development, albeit at a low level (Wang et al., 2016). Both FoxO1 and FoxO3 suppress the development of NK cells. Thus, mTORC2 performs a crucial function of suppressing the hyperactivation of FoxO1 (and potentially FoxO3a), via Akt-mediated phosphorylation and degradation of FoxO1, to allow the iNK cells to progress into the final maturation process. This function of mTORC2-Akt-FoxO1 cascade is validated by the observation that both *Ncr1-Cre -FoxO1^*fl/fl*^* and -*FoxO3*^*fl/fl*^ mice possess much larger CD27^–^CD11b^+^ mNK, a moderately increased CD27^+^CD11b^+^, and comparable CD27^+^CD11b^–^ iNK cell populations (Deng et al., 2015). Thus, the link between mTORC2 and FoxO1 is essential in the early stage of NK cell development. A higher expression of FoxO1 at the immature CD27 single-positive stage is to suppress any untoward expression of transcription factors responsible for the transition into mature NK cells. This is to safeguard an ordered process of gene expression and a faithfully executed NK cell maturation process. One of these transcription factors that is suppressed by FoxO1 is T-bet, which is essential for the terminal maturation and functions of NK cells (Townsend et al., 2004).

T-bet expression and protein levels progressively increase as the iNK cells advance into mature and termNK cells. Studies into the regulation of T-bet expression have provided exciting new insights about the interdependent cooperation and reciprocal suppression by Eomes or FoxO1 (Figure 4). FoxO1 exerts strong transcriptional repression on the *Tbx21* gene during the early NK cell maturation (Deng et al., 2015). While Runx3 (Levanon et al., 2014), E4bp4 (Male et al., 2014; Seillet et al., 2014), Ets-1 (Ramirez et al., 2012), and Tox2 (Vong et al., 2014) support the expression of *Tbx21*, FoxO1 interacts with Sp1 to bind to the *Tbx21* proximal promoter to repress its transcription (Deng et al., 2015). Thus, apart from the positive regulation of Eomes by mTORC1, the negative suppression by mTORC2 plays a crucial role in maintaining the gene signature of immature CD27 single positive NK cells. Further validation of this phenomenon is provided by our laboratory, where we reported that FoxO1 suppresses the transition of iNK to mNK cells through the axis of mTOR2-Akt^*S*473^-FoxO1-T-bet (Yang et al., 2018). Also, FoxO1 suppresses the proliferation of developing NK cells by augmenting the transcription of genes that encode cell-cycle inhibitors (Deng et al., 2015). The continued interplay between FoxO1 and T-bet has been reported even after NK cells are fully matured (Deng et al., 2015). Activation of NK cells with IL-15, IL-2, or IL-12 resulted in the phosphorylation of FoxO1 at Ser^256^, resulting in its inability to be translocated into the nucleus and sequestrated into the cytoplasm for degradation. In concordance, loss of FoxO1 (*Ncr1-Cre-FoxO1^*fl/fl*^*) increased anti-tumor cytotoxicity and the production of inflammatory cytokines, including IFN-γ from NK cells (Deng et al., 2015).

Terminal maturation of NK cells requires Tsc1-dependent negative regulation of IL-15-triggered mTORC1 activation (Yang et al., 2016). IL-15R recruits and activates Jak1/3 that, in turn, phosphorylates and activates Stat5a/b. Besides, activation receptors and IL-15R can initiate the PI(3)K-p110δ/p85α in NK cells (Awasthi et al., 2008; Guo et al., 2008) to link mTORC1 to downstream signaling (Yang et al., 2016). In CD8^+^ T cells, Stat5a/b can initiate transcription of both T-bet and Eomes (Grange et al., 2013), and combined action of T-bet and Eomes at the *Il2rb* promoter region initiates the transcription IL-2Rβ (CD122). The role of Stat5 in activating 4EBP1 has been postulated and thereby linking Stat5 to protein translation. However, an interplay between the IL-15R-Jak1/2-Stat5a/b to IL-15R-PI(3)K-p110δ/p85α-mTORC1 is not well-established. In this context, it is important to note that inhibitors of PI(3)K-mTOR pathway significantly reduced the phosphorylation of Stat5, demonstrating a potential link (Bartalucci et al., 2017). Other cytokine receptors such as IL-12R can also upregulate T-bet expression primarily toward to production of IFN-γ and the role of IL-12 during the development of NK cells is yet to be established (Klose et al., 2014; Zwirner and Ziblat, 2017).

Our recent work has unraveled a link between T-bet and FoxO1 downstream of mTORC2 (Figure 4). Lack of Rictor in NK cells resulted in augmented expression of *FoxO1* and a significant decrease in the transcription of *Tbx21*. Congruently, lack of T-bet significantly augmented expression of *FoxO1*, indicating a reciprocal relationship between these two transcription factors. Importantly, the conditional deletion of FoxO1 in Rictor-deficient mice resulted in the normal expression of T-bet and an increase in the number of termNK cells. These results were substantiated by the fact that T-bet-deficient mice largely phenocopied the developmental defects of NK cells in Rictor-deficient mice and the NK cells from FoxO1-deficient mice contained a significantly reduced level of T-bet. Thus, the suppression and degradation of FoxO1 by the mTORC2-Akt pathway is an essential mechanism for T-bet expression at the transitional stage 2 and 3 of NK cells allowing them to mature and functionally competent.

The ability of T-bet to directly bind to the intronic region and the regulatory region upstream of transcription starting site of *Foxo1* locus strongly supports this notion. Thus, while the Foxo1, which has the highest expression in the CD27 SP subset (Deng et al., 2015), is essential for driving the expression of iNK signature genes, the activation via mTORC2 to shut down FoxO1 function to relieve the repression of T-bet is crucial for the final maturation process. The effector functions of NK cells depend on multiple transcription factors, including T-bet. The anti-tumor cytotoxicity and cytokine production are the major functions of mature NK cells. Lack of Rictor resulted in impaired anti-tumor functions of NK cells *in vivo* (Yang et al., 2018). Several transcription factors regulate the production of IFN-γ and T-bet is one of the major regulators that directly binds to the *ifng* promoter and initiates its transcription (Beima et al., 2006; Miller et al., 2010). This involvement by the fact that T-bet-deficient NK cells have significantly reduced the ability to produce IFN-γ (Townsend et al., 2004; Gordon et al., 2012). Besides, T-bet-deficient NK cells do not sustain the production of IFN-γ (Szabo et al., 2002; Townsend et al., 2004; Gordon et al., 2012). Also, T-bet can bind to *Gzmb*, *prf1*, and *Runx1* promoter regions in NK cells (Townsend et al., 2004; Gordon et al., 2012). T-bet-deficient NK cells are impaired in their anti-tumor effector functions (Townsend et al., 2004; Intlekofer et al., 2005; Gordon et al., 2012). Thus, mTORC2 plays an integral role in NK cell-mediated effector functions.

## Summary and Future Outlook

Mechanistic target of Rapamycin complexes play an essential and complex role in the development and functions of NK cells. The mechanism they utilize to regulate the metabolic reprogramming and transcriptional regulation in NK cells hold promise in identifying novel molecular targets to formulate better immunotherapies. Advanced technologies, including single-cell RNA sequencing, are allowing us to define these mechanisms downstream of mTORC1 and mTORC2. The transcriptional regulations mediated by these complexes are emerging. A novel new pathway via the mTORC2-Akt^*S*473^-FoxO1-T-bet axis regulates the expression of iNK genes during NK cell development. mTORC1-Eomes pathway downstream of IL-15 regulates the early developmental stages of NK cells, while an mTORC2-Akt^*S*473^-FoxO1-T-bet pathway is critical for the terminal maturation of NK cells. Reciprocal activation or repression of Eomes/T-bet and FoxO1/T-bet are two major examples of how mTOR complexes coordinate a complex gene transcription process to mature NK cells. Irrespective of this progress, critical questions remain open. What are the independent roles of mTORC1 and mTORC2 in distinct stages of NK cell development? What roles do mTORC2 play in IL-15-mediated NK cell priming? Are mTORC1 and mTORC2 essential for the downstream signaling of activating receptors? Answers to these questions will help better define the unique roles played by mTOR complexes in human NK cells and help in targeting novel signaling proteins for therapeutic purposes.

## Author Contributions

CY conceived, wrote the manuscript, and generated the figures. SM conceived, wrote, and generated the manuscript, supervised the work, and obtained funding for the work. All authors contributed to the article and approved the submitted version.

## Conflict of Interest

The authors declare that the research was conducted in the absence of any commercial or financial relationships that could be construed as a potential conflict of interest.
